# Future risk projection to engage ‘near-miss’ individuals in lung cancer screening eligibility: an analysis of ILST data

**DOI:** 10.1136/thorax-2024-222098

**Published:** 2025-04-24

**Authors:** Chellan Kumarasamy, Kim Betts, Richard Norman, Annette McWilliams, Emily Stone, David C L Lam, Renee Manser, Paul Fogarty, Henry M Marshall, Stephen Lam, Martin Tammemagi, Kwun M Fong, Sukhinder Atkar-Khattra, Fraser Brims

**Affiliations:** 1 Curtin Medical School, Curtin University, Perth, Western Australia, Australia; 2 Curtin School of Population Health, Curtin University, Perth, Western Australia, Australia; 3 Department of Respiratory Medicine, Fiona Stanley Hospital, Murdoch, Western Australia, Australia; 4 The University of Western Australia, Perth, Western Australia, Australia; 5 Department of Thoracic Medicine and Lung Transplantation, St Vincent’s Hospital Sydney, Sydney, New South Wales, Australia; 6 The University of Melbourne School of Population and Global Health, Melbourne, Victoria, Australia; 7 University of Hong Kong Faculty of Medicine, Hong Kong, Hong Kong; 8 Department of Respiratory Medicine, The Royal Melbourne Hospital, Melbourne, Victoria, Australia; 9 Department of Medicine, The Royal Melbourne Hospital, Melbourne, Victoria, Australia; 10 Department of Internal Medicine, Peter MacCallum Cancer Centre, Melbourne, Victoria, Australia; 11 Internal Medicine Clinical Institute, Epworth HealthCare, Richmond, Victoria, Australia; 12 University of Queensland Thoracic Research Centre, The Prince Charles Hospital, Brisbane, Queensland, Australia; 13 British Columbia Cancer Research Centre, Vancouver, British Columbia, Canada; 14 Department of Medicine, The University of British Columbia, Vancouver, British Columbia, Canada; 15 Department of Health Sciences, Brock University—St. Catharines Campus, St. Catharines, Ontario, Canada; 16 Integrative Oncology, British Columbia Cancer Research Centre, Vancouver, British Columbia, Canada; 17 The University of British Columbia Faculty of Medicine, Vancouver, British Columbia, Canada; 18 Department of Respiratory Medicine, Sir Charles Gairdner Hospital, Perth, Western Australia, Australia; 19 Institute for Respiratory Health, Perth, Western Australia, Australia

**Keywords:** Lung Cancer, Smoking cessation, Clinical Epidemiology

## Abstract

**Introduction:**

Lung cancer risk increases with time, and participants who are initially ineligible for lung cancer screening (LCS) could become eligible later. The aim of this study was to determine the proportion of people (initially ineligible) who may become eligible in a risk model-based LCS programme and the impact smoking cessation could have on this cohort.

**Methods:**

All potential participants for the International Lung Screening Trial aged 55–80 years, ineligible for Low-dose CT screening at baseline (PLCO_m2012_<1.5% 6-year risk), were included. Assuming annual increments of change in age, smoking duration and quit time, and under the assumption of other risk variables being constant, projections of risk were made using the PLCO_m2012_ model from evaluation to the upper age limit of 80 years.

**Results:**

4451 subjects with a median age of 61 (IQR: 57–66) years were included. Assuming no change in smoking status post evaluation, 2239 participants (50.3%) became eligible (PLCO_m2012_≥1.51%) by age 80, with 26.9% and 38.7% of the cohort reaching eligibility by age 70 and 75 years, respectively. Among participants with a baseline risk≥0.6%, 1518 (34.1%) reached eligibility within 10 years of initial evaluation. Smoking cessation after first evaluation can reduce the proportion of individuals who may become eligible for LCS by age 70 from 68.7% to 24.9%.

**Conclusions:**

Future risk projection of eligibility could provide a time window for reassessment of risk on an individual level. It is important to provide smoking cessation services to individuals who are ineligible for LCS at the initial programme contact.

**WHAT IS ALREADY KNOWN ON THIS TOPIC**

- Eligibility for lung cancer screening (LCS) is driven by risk factors such as age and smoking exposures. Currently, risk prediction models have been proposed as a means of identifying a suitable screening population. Utilisation of lung cancer risk prediction criteria for selecting a screening population necessitates applying a strict cut-off, in order to select individuals. As lung cancer risk is known to increase with age and other factors, ineligible individuals who fall below the cut-off value, but are close to it (termed as ‘near-miss’ individuals), are likely to reach eligible levels of risk with time.

**WHAT THIS STUDY ADDS**

- Nearly half of all individuals who were ineligible for LCS under the International Lung Screening Trial are projected to reach screen eligible risk prior to the cut-off age of 80 years, with three-quarters of all ineligible participants reaching eligibility within 10 years of first assessment. Timely reassessment of risk may ensure that individuals receive LCS when eligible, with the appropriate time for reassessment determined by future risk projection.

**HOW THIS STUDY MIGHT AFFECT RESEARCH, PRACTICE OR POLICY**

- As LCS develops from trial settings to programmatic screening, a framework for reassessment of risk and eligibility needs to be considered, especially for ‘near-miss’ individuals. Future risk projection offers a method of identifying ‘near-miss’ individuals and provides a timeframe for reassessment, thereby potentially improving screening uptake, reach and efficiency.

## Introduction

 Lung cancer is associated with high rates of mortality and poor 5-year survival.^1 2^ Low-dose CT (LDCT)-based lung cancer screening (LCS) of targeted high-risk populations has demonstrated significant improvements in mortality.^3 4^ International implementation of national LCS programmes has been slow, with participant eligibility and cost-effectiveness being a major concern for policy makers.^5^,^9^

Eligibility criteria for LCS attempt to identify a targeted population that can most benefit from LDCT screening by balancing the risk of lung cancer with estimated improvements in mortality.^10^,^14^ These eligibility criteria are either categorical, as seen in US Preventive Services Taskforce (USPSTF) 2021 criteria^15^ and Australia’s recently proposed Medical Services Advisory Committee criteria,^16^ or are defined by probabilistic criteria, such as the PLCO_m2012_^17^ and the Liverpool Lung Project Model V.2^18^ prediction models. These models predict future lung cancer incidence to inform potential participant selection into an LCS programme. All selection methods place heavy emphasis on age and smoking as primary drivers of lung cancer risk.^19^

Selection through lung cancer risk prediction models is performed using qualifying thresholds, such as the PLCO_m2012_≥1.51% 6-year risk used in the International Lung Screen Trial (ILST).^20 21^ However, lung cancer risk is dynamic and increases over time, with age and continued smoking as primary drivers.^17^ Engaging individuals who are ineligible for LCS at the time of first contact could improve overall screening uptake in a ‘near-miss’ population, that is, a population close to being eligible, and who may reach the eligibility threshold in future years.

The ILST enrolled 4054 high-risk participants for LDCT lung screening, from seven sites across Australia, Canada and Hong Kong. The aim of the current study was to analyse the ILST respondents who were *ineligible* for screening participation at the initial assessment and evaluate future risk trends and, further, the impact of smoking cessation of the population using projections of PLCO_m2012_ lung cancer risk.

## Methods

### Study population

This post hoc cohort study derived its population from the ILST participants ineligible for screening and included all individuals between 55 and 80 years of age, who had a PLCO_m2012_ risk score<1.51% at initial assessment. This study only considered ineligibility based on PLCO_m2012_ lung cancer risk scores and did not preclude eligibility by other means, such as the USPSTF 2021 criteria.^20 22^ Missing and implausible values in the data were noted to be the result of random human error during data entry. Therefore, as values were assumed to be missing completely at random and had known associations with other variables in the data, Multiple Imputation by Chained Equations (MICE) were chosen as the method to address missing values, facilitated by the ‘MICE’ package in R (V.4.3.1).

### Procedures

The PLCO_m2012_ lung cancer risk model combines 11 variables to estimate an individual’s risk of developing lung cancer within the next 6 years (age, education (the study adjusted for Australian population), body mass index (BMI), presence of COPD/emphysema/chronic bronchitis, personal cancer history, family lung cancer history, ethnicity, smoking status, smoking intensity, smoking duration and time since the individual quit smoking). Projections of future risk assumed COPD status, personal cancer history and family history of lung cancer, education level, as constants from first assessment onward as they were least subject to change over time. Multivariable linear regression was used to assess the relationship between age and BMI in the ILST cohort, accounting for the covariates of smoking duration, education level and sex, due to their influence on BMI and their inclusion in the PLCO_m2012_ risk model. Analysis indicated a risk increase due to BMI with respect to age. However, the additional presence of confounders such as smoking in the cohort indicated a complex nonlinear relationship between age and the other risk factors. Therefore, BMI was assumed constant in order to minimise further assumption, as well as reduce complexity with regard to future implementation.

As predicting individual smoking behaviour was not feasible, projections of risk also assumed no change in the smoking status of former or current smokers, as well as a consistent smoking intensity in current smokers. Annual increases in age, smoking duration and time since quitting smoking were used to calculate projected PLCO_m2012_ risk scores from age at first evaluation to 80 years of age, generating projected risk trends. Annual projections of risk were calculated on an individual basis. To assess the impact of smoking cessation after initial evaluation, we compared the aforementioned assumption of no change in smoking status (continued smoking at current intensity) to an assumption of complete and successful smoking cessation after initial evaluation. This allowed us to estimate the maximum possible benefit that could be achieved by a smoking cessation programme offered to this cohort. This also assumed no relapse after smoking cessation.

### Statistical analysis

Lung cancer risk was estimated using the PLCO_m2012_ risk calculation tool built in R using previously published model predictor beta coefficients.^17^ Lung cancer risk factors and demographic characteristics were reported using contingency tables for categorical variables and descriptive statistics for continuous variables. Linear regression analysis was used to identify correlations between lung cancer risk factors. Differences in proportions of participants eligible by age thresholds of 70, 75 and 80, as well as the proportions of participants eligible before and after successful smoking cessation, at each of the age thresholds, were evaluated using a χ^2^ test.

Subgroup analysis was conducted based on the location of ILST sites. Graphical analysis was employed to compare both participant eligibility across various age thresholds and eligibility with or without smoking cessation, across the screening sites. The difference in the additional screening duration added to the programme with and without smoking cessation was compared using a Wilcoxon signed rank test. All analyses were conducted using R in RStudio V.4.3.1.^23^

## Results

A total of 10 146 potential participants underwent risk assessment for inclusion in ILST, of which 4883 were ineligible due to PLCO_m2012_ risk<1.51%. Of the initial 4883 participants, 207 were excluded due to age (>80 or <55 years), 215 were excluded due to no smoking exposure and a final 10 were excluded due to extensive missing data for nearly all variables (due to duplicate entries). Subsequently, a total of 4451 participants (91.2%) were included in this current study. Missing and implausible values of BMI, smoking intensity (in cigarettes per day) and smoking duration (in years) accounted for ~1.5% of all participant data and were addressed through 50 iterations of imputation using predictive mean matching, generating a single imputed dataset. The majority of the participants were from ILST’s Australian (55.7%) and Canadian (41.6%) screening sites, with a small proportion of participants from Hong Kong (2.7%).

Baseline characteristics of participants are presented in table 1. The median age was 61 (IQR 57–66) years, with a near even distribution of men to women. The population was predominantly composed of white individuals (87.2%), with a small proportion of Asian (9.1%) and smaller groups of Hispanic, black or Native American (<1%) ethnicities. All participants were either current or former smokers; however, the majority of participants had ceased smoking (76.2%, n=3486), with overall median tobacco smoke exposure and smoking duration being 15 (IQR 10–20) cigarettes per day and 27 (IQR 19–35) years, respectively. Of those who had ceased smoking, the median time since cessation was 15 (IQR 1–28) years. At baseline, 8.2% of participants reported having COPD, emphysema or chronic bronchitis. Similarly, family history of lung cancer (16.3%) and a personal history of cancer (9.8%) were not widely prevalent in the cohort.

**Table 1:** Demographic and clinical characteristics of study cohort

Total (n=4451)	Overall median (IQR)/n (%)	Missing n (%)
Age (years)	61 (57–66)	–
Body mass index	26 (24–30)	9 (0.2%)
Smoking status		
Former	3486 (78.3%)	–
Current	965 (21.7%)	–
Duration smoking (years)	27 (19–35)	15 (0.3%)
Smoking quit time (years)	20 (10–31)	8 (0.2%)
Smoking intensity (cigarettes per day)	15 (10–20)	14 (0.3%)
Education level*	4 (2–5)	–
Chronic obstructive pulmonary disorder	366 (8.2%)	–
Personal cancer history	434 (9.8%)	–
Family history of lung cancer	724 (16.3%)	–

Continuous variables as median (IQR), and categorical variables as number (%).

* Education levels in the table correspond to—1: less than high school graduation, 2: high school graduate, 3: post high school training, 4: some college training, 5: college graduate, 6: postgraduate or professional training.

### Change in lung cancer risk

Projected PLCO_m2012_ risk scores demonstrate that around half (50.3%, 95% CI 48.8% to 51.7%; n=2239) of the participants becoming eligible (≥1.51% 6-year risk), prior to the age of 80 years. Restricting eligibility age thresholds to 75 and 70 years reduced the proportion potentially eligible to 38.7% (95% CI 37.3% to 40.2%) and 26.9% (95% CI 25.6% to 28.2%), respectively. Analysis of former smokers who had achieved cessation prior to initial evaluation indicated that only 41% (95% CI 39.3% to 42.6%) achieved risk above screening threshold by age 80, with only 28.1% (95% CI 26.6% to 29.6%) and 15.4% (95% CI 14.2% to 16.6%) reaching eligibility by age 75 and 70, respectively (for sensitivity analysis of future eligibility with differing thresholds of PLCO_m2012_ 6-year risk, see online supplemental table 1).

The proportions of participants eligible at each age threshold maintained a consistent trend across participating sites (for demographic details of participants by location, see online supplemental table 2). Hong Kong was noted to have the highest proportion of participants eligible for screening by age 80 (72.2%, 95% CI 63.3% to 80%), 75 (68.1%, 95% CI 58.9% to 76.3%) and 70 (58%, 95% CI 48.6% to 66.7%), primarily due to higher smoking rates in the subgroup (51.2% of participants were current smokers).

**Figure 1:**
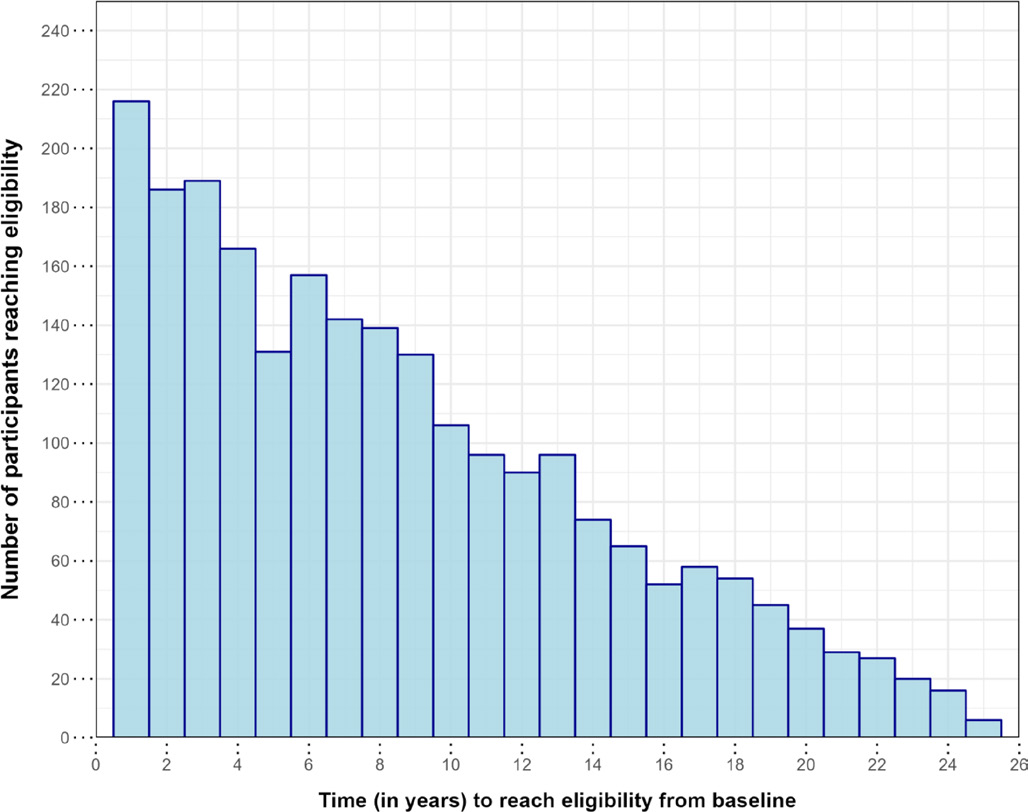
Histogram of years taken to reach eligibility (as per projected risk trends) from baseline assessment.

Of participants eligible by age 80, a majority (69%, 95% CI 67.1% to 71%; n=1546) reached threshold risk within 10 years of baseline assessment (figure 1), and of these, 78.3% (95% CI 76.1% to 80.3%; n=1210) were under the age of 65 at initial evaluation. Even in participants eligible prior to age 70, a majority (59.6%, 95% CI 56.8% to 62.4%, n=714) reached eligibility within 5 years of assessment.

**Figure 2:**
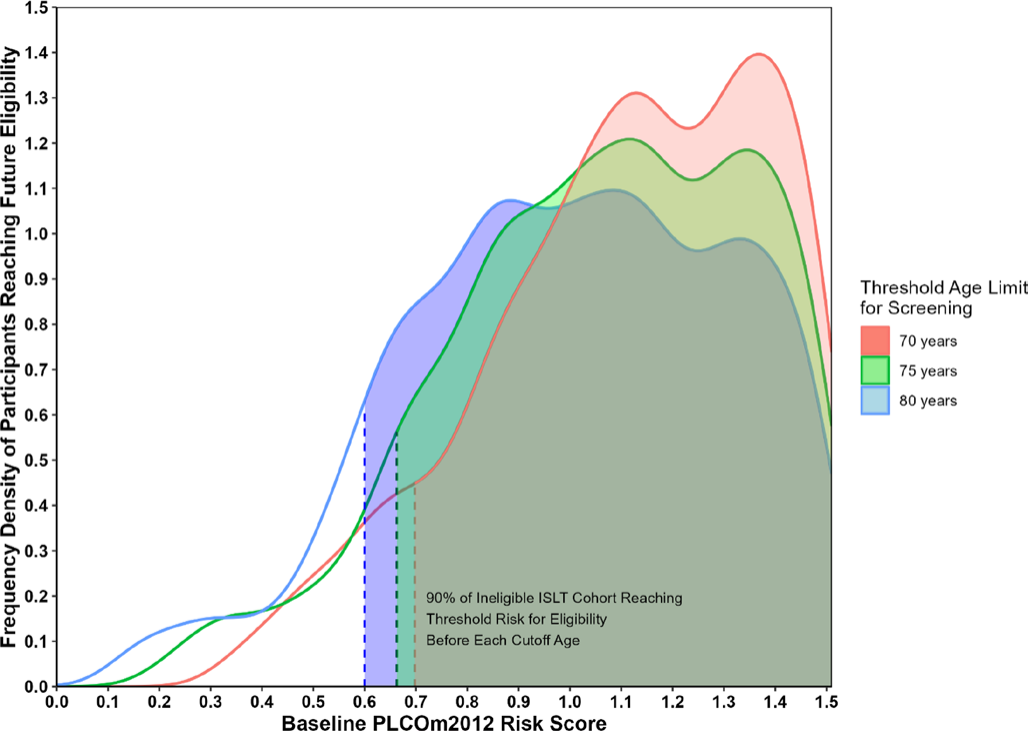
Line plots depicting baseline PLCO_m2012_ risk values at initial evaluation and the corresponding frequency density of participants who are projected to reach threshold risk for eligibility, across three different cut-off age limits for screening (70, 75 and 80 years). The left bounds of the shaded areas indicate the baseline risk values (on x-axis) above which 90% of participants are projected to reach eligibility. ILST, International Lung Screen Trial.

Assessment of the baseline risk values against the proportion of participants potentially eligible beyond each baseline risk value (represented in figure 2 as a density plot with the area under each curve equating to 1 or 100% of the cohort, and the curve highlighting the distribution) demonstrated 90% of the cohort reaching eligibility by ages 80, 75 and 70, when above baseline PLCO_m2012_ risk values of 0.6, 0.66 and 0.7, respectively. This implies that a baseline PLCO_m2012_ risk of between 0.6–0.7 and above accounts for 90% of all potentially eligible participants across all age thresholds. The mean PLCO_m2012_ scores increased across 10-year age groups (from 55 to 80) and were found to be significantly different between the groups (F=5.841, p=0.0029), indicating that the cohort’s overall risk trends significantly towards an increase with age.

### Smoking cessation

**Figure 3:**
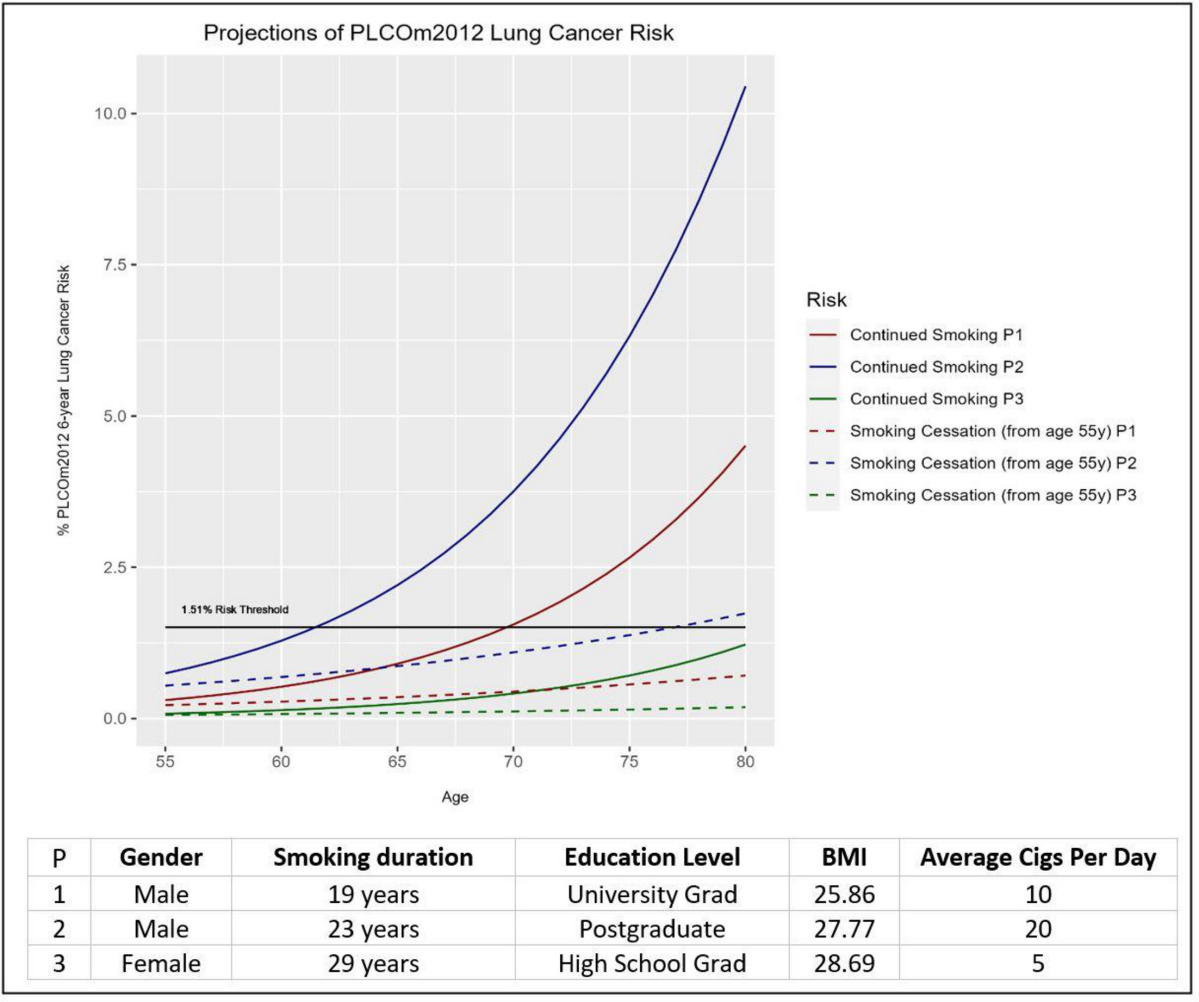
Projections of lung cancer risk change over time with and without smoking cessation, until age 80 for three sample individuals of white ethnicity, aged 55, with no personal/family history of cancer, from the International Lung Screen Trial cohort. BMI, body mass index.

Successful smoking cessation after initial evaluation was found to reduce potential eligibility for screening by significantly reducing the number eligible to 1963 (44.1%, 95% CI 42.6% to 45.5%) individuals prior to age 80 (χ^2^=34.09, p<0.001). Similar significant reductions were observed at age thresholds of 75 (30.9%, 95% CI 29.6% to 32.3%; n=1377) and 70 (17.4%, 95% CI 16.3% to 18.6%; n=775). This results in a 29.7% reduction in the number of annual screening rounds added to a hypothetical programme compared with a no cessation scenario (7586 fewer scans). The reduction in the potential need for screening was observed to be highest at the screening site of Hong Kong (22%–52%), highlighting the benefit of smoking cessation at the time of initial contact in populations with high smoking rates (online supplemental figure 1). Figure 3 gives an example of future lung cancer risk projections to demonstrate the potential impact of smoking cessation on future risk.

## Discussion

This study demonstrates that, among those initially ineligible for LCS in the ILST, over a quarter are projected to reach a high enough lung cancer risk to be eligible prior to age 70, and up to half by age 80. This suggests that current recruitment strategies that assess eligibility only during initial baseline evaluation may miss a large number of individuals who will be eligible for LCS in the future. Current and proposed LCS recruitment strategies at present do not account for projected future risk on eligibility in screening programmes.^5 6 24 25^

In contrast to a clinical trial setting, programmatic screening is not limited to a few screening rounds, with the benefit of LCS being sustained over repeat rounds of screening. It is well known that lung cancer risk increases with age and environmental exposures in addition to continued tobacco smoking.^34 25^,^28^ The implementation and future success of LCS programmes are contingent on targeting a high-risk population to enrich the pretest population and improve healthcare economic gains. In order to contribute to future success, we posit that strategies to engage near-miss participants could be considered.

Projections of future risk also provide a framework for reassessment of ‘near-miss’ individuals in programmatic LCS. Instead of a regular reassessment of risk, individuals could be advised to contact the programme for reassessment closer to the timeframe at which their risk projections suggest that they will reach threshold risk. However, given that this approach to reassessment does not account for any changes in risk stemming from a cancer or COPD diagnosis, or lung cancer in a family member, there would be a need for individuals to contact the programme for risk reassessment if these important changes were to occur.

Additionally, the observation that a majority of the ineligible participants with a baseline PLCO_m2012_ score≥0.6 reach future eligibility potentially provides a simple strategy for identifying those who may benefit from reassessment, which could help improve screening coverage. However, as this lower limit of baseline risk (PLCO_m2012_ risk≥0.6) is specific to the ILST cohort, other LCS programmes may need to modify the lower limit for reassessment based on their target population.

As our study demonstrates, the impact of the proposed framework on recruitment will depend on the upper age cut-off of a programme, which varies across countries, as seen in the USA at age 80 years and the proposed Australian programme cut-off at 70 years.^15 16^ Increasing age can also potentially increase competing comorbidities, but the projected health benefits are beyond the scope of this study.^29 30^

The results indicate that nearly three-quarters of those initially ineligible at baseline reach eligibility within 10 years of first assessment. The projections in this study also support the provision of smoking cessation services at the initial contact, even for individuals who are not eligible for LCS, as the proportion of current smokers who reach future eligibility is significantly higher than former smokers who have already quit smoking. Further, the projected risk after smoking cessation significantly altered the proportion of individuals becoming eligible in the future (figure 3). This positive effect of smoking cessation could also have an impact on the cost-effectiveness of LDCT screening, by lowering the screening eligibility rates and eliminating the need for screening in a large proportion of current smoking individuals.

This study is not without limitations. As the PLCO_m2012_ model was used to generate risk projections, the impact of gender on future risk trends and potential eligibility could not be assessed, as the PLCO_m2012_ does not include sex as a predictor variable. Additionally, all projections of future risk were made under strict assumptions. These assumptions of constancy are unlikely to hold true at an individual level, as smoking status, smoking intensity, adherence to smoking cessation, COPD status, BMI, personal cancer diagnoses, family lung cancer diagnoses and even education level are subject to variation. However, while change in these variables could be addressed via a simulated modelling approach, a predictive model of future risk developed on ILST data would require additional recalibration before being applicable to other target screening populations, which adds complexity to the screening process. The aim of the study was a simple and pragmatic approach to reassessment of risk in programmatic screening, which could be incorporated under existing or proposed LCS frameworks. This approach brings with it assumptions which may not hold true at the individual level, but allow broad applicability across populations. While these limitations exist, they could be ameliorated through a flexible framework that allows for individual reassessment of risk when events that impact risk occur.

Using data from the large-scale prospective ILST study, this study has demonstrated that there is an opportunity for LCS programmes to identify near-miss participants to improve screening uptake. Additionally, integrating preventive interventions such as smoking cessation at the time of initial contact has additional benefits in reducing lung cancer risk.

## Supplementary material

10.1136/thorax-2024-222098 online supplemental file 1

10.1136/thorax-2024-222098 online supplemental figure 1

## Data Availability

Data are available on reasonable request.
