# Supplementary material for: Future risk projection to engage ‘near-miss’ individuals in lung cancer screening eligibility: an analysis of ILST data
Source: Thorax. 2025 Apr 24;80(8):e222098. doi: 10.1136/thorax-2024-222098 (PMC12322426; doi:10.1136/thorax-2024-222098)
Supplement: online supplemental file 1 [file thorax-80-8-s001.pdf]

Supplementary Table 1. Sensitivity analysis assessing the impact of varying the threshold PLCOm2012 risk for screening, on future eligibility of participants

n (%)	PLCO$_{m2012}$ Risk Threshold for Lung Screening					
	≥1.25% PLCO$_{m2012}$ 6-year risk			≥1.75% PLCOm2012 6-year risk		
**Age (years)**	**All Participants**	**Former Smoking**	**Current Smoking**	**All Participants**	**Former Smoking**	**Current Smoking**
**Eligible before 80**	2610 (58.6%)	1769 (50.7%)	841 (87.2%)	1936 (43.5%)	1146 (32.9%)	790 (81.9%)
**Eligible before 75**	2098 (47.1%)	1338 (38.4%)	760 (78.8%)	1434 (32.2%)	704 (20.2%)	730 (75.6%)
**Eligible before 70**	1567 (35.2%)	864 (24.8%)	703 (72.9%)	923 20.7%)	303 (8.7%)	620 (64.3%)

Supplementary Table 2. Demographic and clinical characteristics of study cohort by location. Table describes continuous variables as Median (IQR), and categorical variables as Number (%).

Total (n = 4451)		Overall Median (Inter Quartile Range) / n (%)						
**Location**		**Vancouver**	**Perth**	**Brisbane**	**Sydney**	**Melbourne**	**Hong Kong**	**Epworth**
**Number of Participants**		**1851**	**1080**	**552**	**417**	**325**	**119**	**101**
**Age (years)**		61 (57 – 66)	62 (58 – 69)	60 (57 – 65)	60 (57 – 65)	60 (57 – 65)	60 (57 – 63)	60 (57 – 65)
**Body Mass Index**		26.8 (24.1 – 30.3)	27.5 (24.3 – 30.7)	27.5 (24.9 – 31.1)	26.6 (23.6 – 29.9)	26.6 (23.6 – 30.4)	24.2 (23.6 – 30.4)	26.9 (24.5 – 29.4)
**Smoking Status**	**Former**	1429 (77.2%)	965 (89.4%)	409 (74.1%)	300 (71.9%)	249 (76.6%)	58 (48.7%)	70 (69.3%)
	**Current**	422 (22.8%)	115 (10.6%)	143 (25.9%)	117 (28.1%)	76 (23.4%	61 (51.3%)	31 (30.7%)
**Duration Smoking (years)**		29 (22 – 36)	20 (12 – 30)	27 (18 – 36)	28 (18 – 37)	26 (18 – 35)	35 (31 – 40)	30 (19 – 37)
**Smoking Quit Time (years)**		19 (10 – 27)	27 (16 – 36)	20 (10 – 31)	20 (8 – 30.3)	18 (10 – 29)	9.5 (4.3 – 15.5)	12.5 (5 – 28.3)
**Smoking Intensity (cigs per day)**		18 (10 – 20)	15 (9 – 20)	15 (10 – 20)	15 (10 – 20)	15 (10 – 22)	15 (10 – 20)	15 (10 – 20)
**Education Level***		4 (3 – 5)	3 (2 – 5)	3 (2 – 5)	3 (2 – 5)	4 (2 – 5)	2 (1 – 2)	4 (2 – 5)
**Chronic Obstructive Pulmonary Disorder**		146 (7.9%)	63 (5.8%)	64 (11.6%)	37 (8.9%)	41 (12.6%)	9 (7.6%)	6 (5.9%)
**Personal Cancer History**		150 (8.1%)	131 (12.1%)	63 (11.4%)	42 (10.1%)	28 (8.6%)	5 (4.2%)	12 (11.9%)
**Family History of Lung Cancer**		349 (18.9%)	145 (13.4%)	80 (14.5%)	75 (18%)	48 (14.8%)	13 (10.9%)	13 (12.9%)
