# Supplementary material for: Future risk projection to engage ‘near-miss’ individuals in lung cancer screening eligibility: an analysis of ILST data
Source: Thorax. 2025 Apr 24;80(8):e222098. doi: 10.1136/thorax-2024-222098 (PMC12322426; doi:10.1136/thorax-2024-222098)

Supplementary Figure 1. Proportions of participants potentially eligible for screening, compared between each age threshold (70, 75 and 80), and with or without smoking cessation, across all International Lung Screen trial (ILST) screening sites.

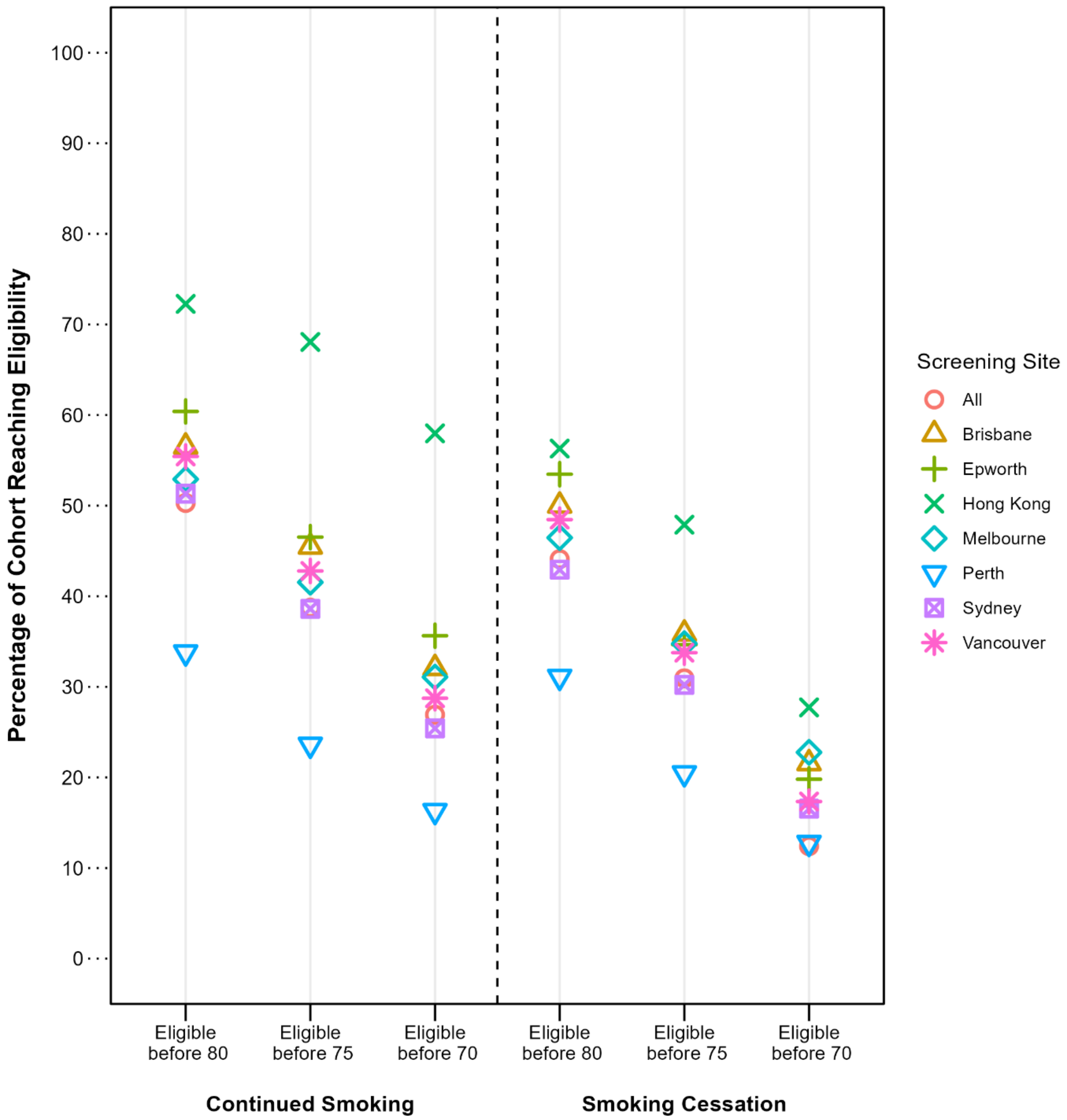

Supplement: online supplemental figure 1 [file thorax-80-8-s002.pdf]
